# Clinical and imaging phenotypes

**DOI:** 10.1093/eurheartjsupp/suaf096

**Published:** 2026-01-06

**Authors:** Giovanni Benfari, Davide Margonato, Marco Metra, Antoni Bayes-Genis, Raffi Bekeredjian, Denisa Muraru, Maurice Enriquez-Sarano

**Affiliations:** Section of Cardiology, Department of Medicine, University of Verona, Verona, Italy; Cardiovascular Imaging Unit, San Raffaele Hospital, Milan, Italy; Department of Cardiology, ASST Spedali Civili and University of Brescia, 25123, Brescia, Italy; Institut del Cor, Hospital Universitari Germans Trias i Pujol, CIBERCV, Badalona, Spain; Department of Cardiology, Robert Bosch Hospital, Stuttgart, Germany; Department of Cardiology, Istituto Auxologico Italiano, IRCCS, Milan, Italy; Department of Medicine and Surgery, University of Milano-Bicocca, Milan, Italy; Cardiovascular Imaging Research Center, Minneapolis Heart Institute and Foundation, Minneapolis, MN, USA

**Keywords:** Tricuspid regurgitation, Severity grading, Clinical phenotypes, Echocardiography, Cardiovascular imaging, Outcome

## Abstract

Functional/Secondary tricuspid regurgitation (STR) accounts for over 85% of clinically significant tricuspid regurgitation (TR) and is associated with adverse prognosis and impaired quality of life. Advances in percutaneous tricuspid valve (TV) interventions underscore the need to differentiate TR aetiologies, mechanisms, and phenotypes. STR is subdivided into atrial (A-STR), caused by right atrial dilation and tricuspid annular enlargement without significant leaflet tethering, and ventricular (V-STR), resulting from right ventricular dilation/dysfunction with leaflet tethering. A-STR, increasingly prevalent with ageing and atrial fibrillation, typically presents with preserved right ventricular function, whereas V-STR reflects more advanced disease, is associated with RV dysfunction and, often, left ventricular systolic dysfunction and remodelling and/or left-sided valve disease, carrying higher mortality, compared with A-STR. Cardiac implantable electronic device (CIED) related TR is emerging as a distinct entity, while organic-TR arises from intrinsic structural abnormalities of the valve apparatus. Echocardiography, particularly three-dimensional imaging, is essential for accurate phenotyping and helps in procedural planning. Medical therapy remains primarily symptomatic, with diuretics as first-line therapy and targeted treatment of underlying cardiac pathology. In A-STR, rhythm control strategies, including catheter ablation for atrial fibrillation, may reverse annular remodelling. Surgical repair, preferably annuloplasty, is recommended in selected patients, often when concomitant left-sided surgery is needed. Transcatheter edge-to-edge repair offers a safe and increasingly used alternative, providing symptomatic improvement. The effects on outcomes are likely dependent on the time of intervention and the STR phenotype.

## Introduction to TR epidemiology, aetiologies, and mechanisms

The growing clinical interest in TR as a significant health burden—together with the development of percutaneous tricuspid valve (TV) procedures over the past decade—has emphasized the importance of distinguishing not only between different TR aetiologies, but also between different STR mechanisms, since, as in all valvular heart disease, aetiology and mechanism are distinct concepts. Moreover, the clinical context in which TR occurs adds considerable heterogeneity to both clinical presentation and prognosis.’^1-5^ An accurate classification is essential to guide the selection of the most appropriate intervention and maximize procedural success in individual patients.

**Figure 1:**
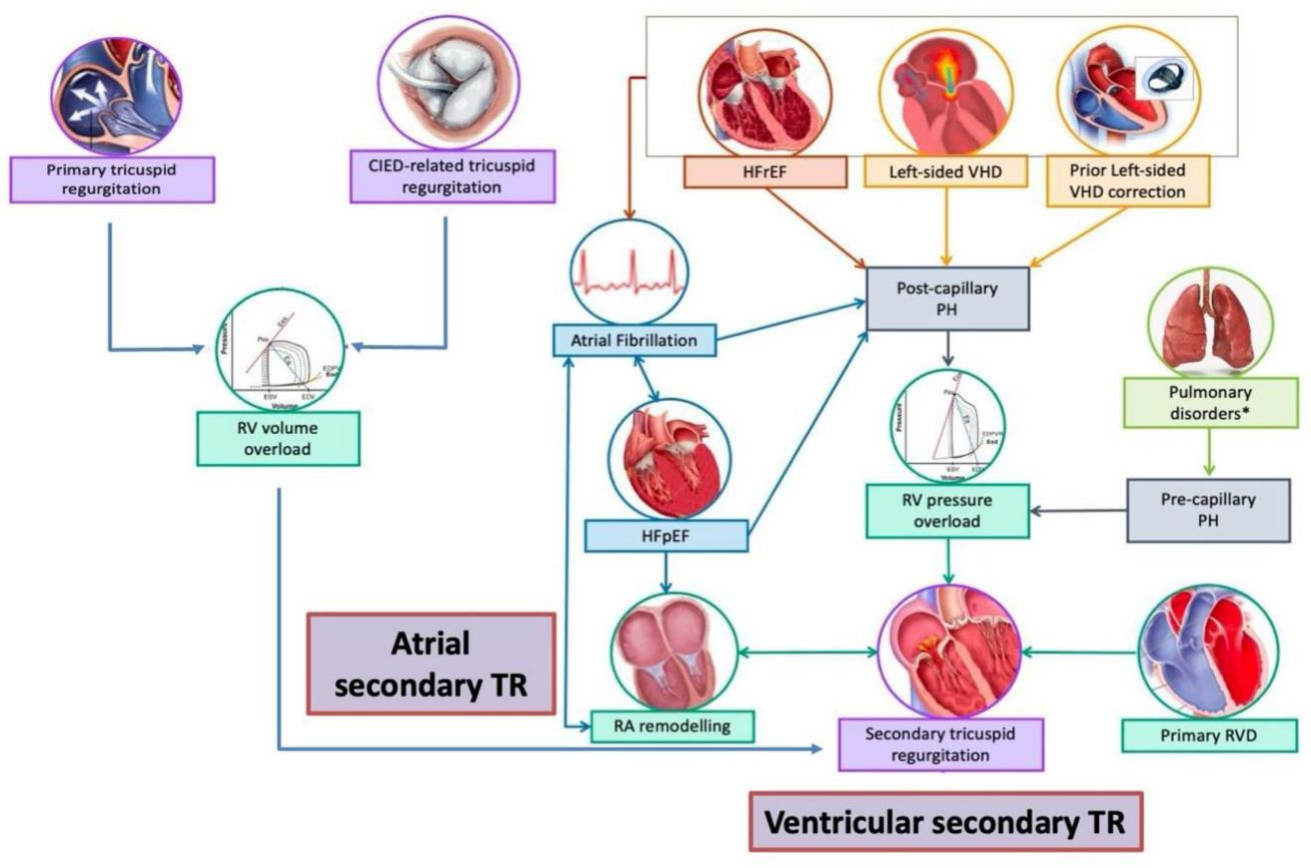
Mechanisms and consequences associated to tricuspid regurgitation phenotypes. In atrial secondary tricuspid regurgitation (TR), the key drivers are atrial fibrillation, heart failure with preserved ejection fraction (HFpEF), and consequent right atrial (RA) remodeling, which promote tricuspid annular dilation and regurgitation. In contrast, ventricular secondary TR develops primarily from right ventricular (RV) pressure overload and structural RV disease. This is often a consequence of post-capillary pulmonary hypertension (PH) due to left-sided heart disease, or less frequently, pre-capillary PH, both of which lead to RV remodelling and dysfunction. Additionally, primary TR and CIED-related TR contribute directly to RV volume overload, further exacerbating the pathophysiological cascade. (Adapted with permission from Adamo et al.^6^).

Most clinically significant TR cases—estimated above 85%—are functional/secondary in nature. Two main subtypes of STR have been identified (*Figure 1*).^7-9^

### Atrial secondary tricuspid regurgitation (A-STR)

Atrial secondary tricuspid regurgitation (A-STR), is by far the most frequent (up to 85% of the STR cases), primarily driven by right atrial (RA) dilation and dysfunction, leading to tricuspid annular (TA) enlargement and loss of its sphincter-like contraction, without primary leaflet pathology or significant valve tethering.^10-14^ A-STR typically develops in patients with long-standing atrial fibrillation (AF), preserved right ventricular (RV) size and function, and heart failure (HF) with preserved left ventricular ejection fraction (HFpEF).^14,15^ Due to the aging population and rising AF incidence,^16-18^ A-STR represents a growing proportion of all TR subtypes.

### Ventricular secondary tricuspid regurgitation (V-STR)

The second type of STR is the ventricular secondary tricuspid regurgitation (V-STR). It was initially thought to be the predominant phenotype, but it represents a minority, comprising one-quarter to one-fifth of cases. V-STR is typically characterized by leaflet tethering, secondary to papillary muscle displacement and geometric distortion of the RV.^19^ It is more common in the presence of concomitant left-sided heart disease, including either HF with reduced left ventricular ejection fraction (HFrEF) and/or left-sided valve disease. Pulmonary hypertension and RV dysfunction are the hallmarks of this clinical presentation.^6^ V-STR is undoubtedly associated with a more advanced disease state and poorer clinical outcomes, suggesting it might be seen as a later and more severe stage in the continuum of HF and/or left heart valve disease.^20^ Of note, at the time of severe TR diagnosis, it is often challenging to define whether RV dilation was the initial trigger of the regurgitation, or vice versa. The actual role of these two mechanisms could better be established based on repeated evaluations over time.^21,22^

This pathophysiological grouping represents a conceptual shift—moving beyond simply identifying the aetiology of TR (organic/primary vs. functional/secondary) to understanding which cardiac chamber is primarily responsible for STR. *Table 1* summarizes the specific features of A-STR and V-STR.

**Table 1:** Overview of secondary tricuspid regurgitation phenotypes, CIED related TR and organic TR

	Secondary TR		CIED-Related TR	Organic TR
	V-STR	A-STR		
**Epidemiology**	The most commonly encountered.> in elderly with atrial fibrillation and right atrial dilation;> in women.	Common in left-sided heart disease, pulmonary hypertension, RV dysfunction; often secondary to LV disease	Occurs in 10–40% of patients with cardiac implantable electronic devices (pacemakers, ICDs).	Less common overall; prolapsing leaflets, rheumatic disease, infective endocarditis, carcinoid, congenital anomalies
**Pathophysiology**	RA dilation (often from long-standing AF) enlarges tricuspid annulus without significant RV dilation/tethering	RV dilation and/or dysfunction leads to tricuspid annular dilation and leaflet tethering; functional/secondary but with ventricular-driven geometry changes	Device leads impinge on leaflets, cause tethering, perforation, or fibrosis; may also cause leaflet entanglement	Structural leaflet pathology from congenital, inflammatory, infectious, or infiltrative processes
**Clinical Presentation**	Insidious onset with right HF symptoms; frequently in patients with preserved RV function and AF	Often asymptomatic early; may present with right-sided HF signs (oedema, ascites, hepatomegaly) in advanced disease	Can be asymptomatic initially; progression leads to right HF symptoms; sometimes associated with worsening after device implantation	Variable; may present acutely (i.e.: infective endocarditis) or chronic.
**Outcome**	Generally better tolerated if RV function preserved, but can progress to RV failure	Poor prognosis if severe, especially when associated with advanced LV/RV dysfunction; high mortality	Associated with worse survival compared to similar patients without TR; may improve if device cause corrected	Prognosis depends on aetiology and treatment; infectious forms have high acute mortality.
**Intervention**	Manage AF and RA dilation; surgery or transcatheter repair if symptomatic and severe	Treat underlying left-sided disease and pulmonary hypertension; surgery/transcatheter repair if severe and symptomatic	Could require lead extraction/repositioning; surgical or transcatheter repair if TR persists or is severe	Surgery (repair or replacement) indicated if severe or in combination with other valve surgery; targeted treatment of underlying cause

### Cardiac implantable electronic device (cIED)–related TR

In addition to A-STR and V-STR, cardiac implantable electronic device (cIED)–related TR represents a growing clinical entity, particularly with the increasing use of pacemakers, implantable cardioverter-defibrillators, and cardiac resynchronization therapy. cIED-related TR may occur through several mechanisms, including direct leaflet impingement or perforation by the lead, entanglement in the subvalvular apparatus, or fibrotic adherence of the lead to the tricuspid valve apparatus, ultimately impairing leaflet coaptation and valve function. Although it shares features with STR, cIED-related TR has unique procedural considerations, especially when planning percutaneous interventions, as the presence of the lead may complicate device deployment or necessitate extraction.^19,23^

### Primary (or organic) TR

Conversely, primary (or organic) TR is caused by intrinsic abnormalities of the tricuspid valve leaflets, chordae, papillary muscles, or annulus. Aetiologies include rheumatic heart disease, infective endocarditis, myxomatous degeneration, carcinoid heart disease, radiation-induced valvulopathy, and congenital malformations such as Ebstein anomaly. In organic TR, the valve apparatus itself is structurally abnormal, and the regurgitation mechanism is independent of right atrial or ventricular dilatation. Surgical or transcatheter repair strategies for organic TR must therefore address the underlying morphological lesion directly, and outcomes are often determined by the extent of associated right heart remodelling and comorbid conditions.^19,24^

cIED-related TR and organic TR will be briefly presented in the second part of this dissertation and are included in *Table 1* along with STR phenotypes.

## STR clinical presentation and outcome

The clinical presentation of TR is frequently insidious, with symptoms developing in the late stage of the disease despite progressive chamber dilation. When present, signs and symptoms might include fatigue, lower limb oedema, ascites, malnutrition, renal failure, and other manifestations of right-sided heart failure, with no specific clues across TR aetiologies.^25^ Furthermore, in many patients, A-STR coexists with atrial secondary mitral regurgitation, a condition which contributes to exercise intolerance and recurrent HF admissions.^26^

It is important to underline that TR is characterized by a markedly variable clinical presentation, reflecting the heterogeneity of causal contexts and the possible coexistence of different heart failure phenotypes. Accordingly, while in some patients it manifests as isolated right-sided congestion, in others it represents the epiphenomenon of advanced biventricular dysfunction, often accompanied by PH and systemic venous stasis. In this complex spectrum, it is of outmost importance to categorize patients risk profile and predict long-term prognosis with *ad hoc* clinical scores: recently, the TRISCORE has emerged as a valuable instrument to integrate such variability, offering a structured approach to risk stratification and facilitating a more individualized prognostic assessment in different clinical scenarios^6,27,28^

Growing evidence recently emerged depict the typical clinical profile of A-STR patients: older age, more often female, with multiple associated comorbidities often represented by arterial hypertension, HFpEF, AF and chronic kidney disease.^29-31^ A bidirectional relationship exists between A-STR, AF and HFpEF so that the risk to develop each of these conditions increases once one of them is present.^32,33^

Notably, despite the relevant burden of concomitant diseases, observational studies indicate that moderate TR is independently associated with excess mortality, even after adjusting for major comorbidities and ventricular function.^29,31,34,35^

Overall, V-STR carries markedly worse long-term survival than A-STR: 10-year survival ∼50% vs. 80% for A-STR; and V-STR independently predicts mortality with a hazard-ratio of 2.9 vs. A-STR.^36^

The risk factors for developing/progressing secondary TR have long been described, and include age, atrial fibrillation, pacemaker/ICD leads, left-sided heart disease, and female sex—factors frequently present in V-STR.^37^

Among patients with isolated TR, an aetiologic outcome model identified V-STR substrates and comorbidities as key mortality predictors, enabling risk-score stratification.^24^

## Echocardiographic grading and pathophysiological consideration in V-STR and A-STR

A comprehensive TR assessment requires a multiparametric, multimodality approach, emphasizing both TV anatomy and RV morphology, despite the challenges posed by the RV’s complex geometry.

Echocardiography remains the first-line imaging modality for evaluating the aetiology, anatomy, and severity of TR. However, this valvular disease is uniquely dynamic: its severity may fluctuate over time, influenced by medical therapy, intravascular volume shifts, respiratory changes and recurrent heart failure decompensations, posing additional challenges for both diagnosis and management.^38^ As temporary response to medical therapy may be misleading, and significant TR present an inherent risk of recurrence, particularly in patient with tricuspid annulus dilatation, precise quantification with echocardiography is pivotal, and assessments should ideally be performed with the patient in an euvolaemic state, during quiet respiration, and—especially in arrhythmic rhythms—averaged on at least 5 cardiac cycles.^10^

The European Society of Cardiology and the European Association of Cardiovascular Imaging recommend a three-grade classification of TR severity, complemented by an expanded five-grade system—severe (3+), massive (4+), and torrential (5+)—for patients undergoing transcatheter interventions.^39^ This expanded classification has clinical value, as even a one-grade reduction is associated with improvements in functional status and quality of life.^40^

### Echocardiography in A-STR and V-STR

In A-STR, tricuspid annular dilation is often asymmetric, predominantly in the antero-posterior dimension, displacing commissures and impairing leaflet coaptation.^12,41^ Due to the complex non-planar shape of the TA, three-dimensional (3D) echocardiography has been pivotal in characterizing these geometric changes and identifying the subtype-specific anatomical and functional prerogatives of A-STR.^11,41,42^

Patients with A-STR typically present with TA dilatation (end-diastolic absolute or indexed dimension ≥40 mm or >21 mm/m2, respectively) and contractile dysfunction, in the absence of significant leaflet tethering (usually <8 mm) and with limited RV dilatation, of its basal portion only (RV basal end- diastolic diameter > RV mid-end-diastolic diameter).^43-45^ RA size is enlarged, often accompanied by left atrial and mitral annular dilation in chronic AF due to biatrial remodelling. Of note, the TA is considered more prone to dilatation compared to the mitral annulus due to its lack of a significant fibrous component except where the leaflets are connected to the central fibrous body, as its structure is mostly represented by fibroadipose tissue; moreover, the RA wall is thinner than the left atrial wall, making it more susceptible to increased intra-atrial pressures.^46,47^

Of note, TR severity does not always correlate with the degree of annular dilation, as adaptive leaflet growth may partially compensate for annular remodelling in some patients, increasing the area of leaflet coverage.^48,49^

In V-STR, the RV frequently exhibits spherical or elliptical remodelling with increases in longitudinal and mid-transverse dimensions. This altered geometry displaces and reorients papillary muscles, resulting in leaflet tethering. Anatomical parameters such as tricuspid annular area, tethering distance, tenting height, and volume are all significantly increased in V-STR compared to A-STR.^14^

### The importance of a quantitative approach

Quantifying tethering (distance, area, volume), coaptation patterns, and RA size provides critical insights into STR pathophysiology and its subtypes. Standard 2D echocardiography often fails to capture the annulus’s complex 3D, nonplanar shape and inter-individual variability. In contrast, 3D echocardiography offers a more accurate and reproducible evaluation.^38^

Functional parameters like TAPSE and the S′ wave are used to estimate RV systolic function, although they are preload-dependent and reflect only the longitudinal shortening of the RV. Due to 2D limitations in assessing RV volumes, 3D echocardiography is preferred to measure RV volumes and ejection fraction, which correlate well with those obtained by cardiac MRI (10.1093/ehjci/jev309). When 3D imaging is unavailable, RV fractional area change and RV longitudinal strain are useful alternatives.^50^

As highlighted by Florescu et al., the complex geometry of the RA, RV, and tricuspid annulus is better represented in 3D imaging. Its superiority is underscored by findings that 31% of healthy individuals were misclassified as having RV dilation based on linear dimensions, while none were misclassified when age- and sex-adjusted 3D reference volumes were used.^14^

Among qualitative parameters, colour Doppler flow is the most immediate tool and is effective for identifying small TR jets. However, its accuracy diminishes in more severe TR due to low right heart pressures, which may cause underestimation.

A-STR is particularly prone to underestimation because its regurgitant jet velocity is lower than in ventricular phenotypes, resulting in smaller colour Doppler jet areas and less striking signals despite significant disease. Moreover, the right ventricle is usually smaller in A-STR, which reduces the apparent chamber enlargement typically used in severity grading. The frequent coexistence of atrial fibrillation adds further complexity, as irregular RR intervals create beat-to-beat variability that complicates accurate quantification.^51^

Vena contracta (VC) width, measured in the apical four-chamber view and averaged over 2–3 cycles, is another key parameter. A VC >7 mm suggests severe TR, while <6 mm may indicate mild/moderate regurgitation. Yet, due to the elliptical shape of the regurgitant orifice and variability in valve anatomy, a single linear measurement may not reflect true severity.^52^

Although long established and widely applicable, quantitative methods such as PISA method present additional challenges. In V-STR, the regurgitant orifice is rarely flat, and tethering often creates wedge-shaped orifices. Furthermore, the non-perfectly-hemispheric nature of flow convergence zones can lead to ∼30–35% underestimation of EROA.^53^ Timing also matters, as TR—like mitral regurgitation—is temporally dynamic and can be over- or underestimated depending on when PISA is measured. Variability in leaflet morphology may also create crescent- or stellate-shaped orifices, making the PISA shell more hemi-ellipsoid than hemispherical, further complicating surface area estimation. In V-STR with significant tethering, correction of the PISA method can be performed by accounting for leaflet angle and low systolic velocity, which improves the association between volumetric quantitation and outcome.^54^

An additional grade of complexity may arise from sex-specific differences in TR-related risk. Women appear to experience comparable risk at lower EROAs and regurgitant volumes, in conjunction with smaller RV volumes, higher ejection fraction, and reduced TA dimensions. In this setting, measuring regurgitant fraction—which normalizes regurgitant volume to forward stroke volume—may become a reliable option across sexes and varying chamber sizes.^55^

Moreover, several studies have demonstrated the feasibility of 3D colour Doppler planimetry of the vena contracta area (VCA) using both TTE and TOE. Integrating such advanced 3D techniques into routine assessment enables more accurate TR grading, prevents underestimation of disease severity, and supports earlier, better-targeted intervention.^56^

Finally, it is essential to remark that quantitative TR assessment should integrate a classical multiparametric approach as advocated by current guidelines vena contracta width, hepatic vein flow, effective regurgitant orifice area, regurgitant volume and fraction^39^; 3D planimetry of the vena contracta and direct annular dimension measurements through multimodality imaging are particularly valuable for procedural planning in transcatheter repair.^57,58^ In addition, the role of advanced imaging techniques to further delineate the specific remodelling pattern of each TR phenotype and their implication with regard to the type and timing of significant TR treatment is highly promising but to be fully defined yet^59^

## Management considerations for STR

### Medical therapy

No pharmacological therapy specifically targets either A-STR or V-STR, as it remains focused on symptom relief and euvolaemia maintenance.

Hence, it is primarily based on loop diuretics as first choice to target volume overload, with careful monitoring of renal function and electrolytes. Treatment of concomitant left-sided disease follows established guidelines, including aggressive management of comorbidities such as systemic hypertension and HF to mitigate progression of HF and RA progressive remodelling. When concomitant HFpEF or HFrEF are diagnosed, medical therapy includes sodium glucose cotransporter 2 inhibitors (SGLT2i) and possibly nonsteroidal mineralocorticoid antagonists (MRAs). A reduced or mildly reduced LV ejection fraction is also an indication to angiotensin receptor neprilysin inhibitors (ARNI), or angiotensin converting enzyme inhibitors (ACEi) or receptor blockers (ARBs) and beta-blockers, when tolerated.

Management of secondary TR also involves assessing pulmonary hypertension, left ventricular function, and controlling atrial fibrillation, with right heart catheterization recommended in every case of suspected pulmonary hypertension. These treatments may ease symptoms but do not halt progression in advanced V-STR.

### AF ablation

A-STR often develops in the context of chronic AF and rhythm control is therefore central. In particular, in the recent years successful catheter ablation, resulting in sinus rhythm maintenance, has been demonstrated to improve the severity of TR, suggesting a possible induction of reverse positive atrial and tricuspid valvular remodelling, and highlighting the crucial role of appropriate TA sphincteric contraction to maintain tricuspid valve continency in the absence of irregular cardiac rhythm.^60-62^ Thus, in patients with severe A-STR, if successful AF ablation can be expected, this approach should be prioritized before considering valvular intervention to address TR. Conversely, since at least moderate-to-severe TR has been linked to AF recurrence after successful catheter ablation, transcatheter treatment of TR might also be considered prior to the ablation procedure.^63^

### Surgery

Surgery remains the gold standard for eligible patients. Current guidelines give a Class I recommendation for surgical treatment of severe TR in patients undergoing left-sided valve surgery. Surgery should also be considered in moderate TR with significant tricuspid annular dilatation (≥40 mm), or in isolated severe TR before RV function deteriorates. Valve repair is preferred over replacement due to better long-term outcomes. Among repair techniques, ring annuloplasty offers superior durability and lower recurrence rates of TR compared to suture-based repairs like De Vega. However, rigid rings can increase the risk of suture dehiscence compared to more flexible devices.^64-67^

Surgical AF ablation may be considered, particularly when combined with surgery for concomitant valve disease is indicated.^68^ Interestingly, surgical AF ablation even during concomitant MV intervention is associated with reduced TR progression and right-heart maladaptive remodelling.^69^ However, isolated surgery for A-STR is uncommon due to high operative risk and late presentation. In selected low-risk patients with severe, symptomatic A-STR and preserved RV function, early intervention before irreversible RV or end-organ damage may be advantageous, and annuloplasty techniques restoring TA geometry and function are usually preferred over replacement when feasible.^70^ Accordingly, current guidelines emphasize the role of TV intervention during concomitant left-side heart surgery even for nonsevere TR, in the presence of TA dilatation^64^

### Transcatheter therapies

The development of transcatheter therapies has introduced a promising alternative for patients with severe TR who are considered high-risk or ineligible for surgery. The TRILUMINATE pivotal trial assessed the safety and efficacy of transcatheter edge-to-edge repair (T-TEER) using the TriClip device compared to standard medical treatment. The study confirmed that T-TEER is safe and significantly reduces TR severity. Most notably, it led to a marked and sustained improvement in patients’ quality of life, as evaluated by the Kansas City Cardiomyopathy Questionnaire (KCCQ).^40^ Of note, it included mostly patients with A-STR (preserved biventricular ejection fraction on average, 87% with AF, dilatated TA and RA).^71^ Similar results were more recently demonstrated in the TRI-FR trial, specifically designed to include isolated STR patients.^72^ Both TRILUMINATE Pivotal and TRI-FR trial recruited patients at relatively low risk and, although they both met their primary endpoint, a combined one including both quality of life, HF hospitalizations and mortality, failed to reach a significant reduction in either HF hospitalizations or mortality alone.^71,72^ Thus, inclusion of a higher risk patient population, such as with having a recent HF hospitalizations among inclusion criteria, may be necessary to show significant results also on outcomes.^73,74^

Consistently, a significant reduction in HF hospitalizations was shown in the two-years extension of the TRILUMINATE Pivotal trial.^75^

This supports the concept of a ‘therapeutic window’—a critical time frame during which intervention may still alter the disease trajectory. Supporting this notion, data from the EuroTR registry indicated that T-TEER was associated with improved 1-year survival only in patients with intermediate-stage disease, not in those with either early or advanced disease stages.^31,40,76^

## Cardiac implantable electronic devices-related TR

Despite the renewed attention on the epidemiological burden and independent prognostic role of TR emerged in the last decade, patients with TR and a cardiac implantable electronic device (CIED) remain poorly understood due to the complexity of the frequent left-sided disease, lead-leaflet uncertain interaction and intricate physiology. The prevalence of CIED-related TR is still under debate, with an initial reported frequency of TR following CIED implantation varying from 7 up to 45%.^23,77,78^

These conflicting reports are not surprising, as many studies were based on 2D transthoracic echocardiography assessment of the TV, which presents several limitations on the evaluation of the TV apparatus, on the visualization of the lead crossing the annulus and on the specific mechanism leading to CIED-induced TR. However, as CIED-related TR is increasingly identified as an independent clinical entity, the presence of a lead across the TV and the multiple mechanisms of TR in the presence of a CIED have led investigators to reclassify CIED-related TR as a separate aetiologic entity with its own dedicated evaluation and management.

Three-dimensional echocardiography allows a precise and dynamic visualization of the spatial relationship of the CIED lead with TV leaflets, for understanding the underlying mechanism of TR: direct lead adherence to the leaflet or to the subvalvular structures, impingement causing leaflet malcoaptation, leaflet perforation, or direct damage of the TV apparatus after lead extraction.^23,79-81^

In addition, cardiac computed tomography may provide complementary information on the TV leaflets’ relationship with the transvalvular lead throughout the cardiac cycle, allowing a complete evaluation of the TV apparatus and of the right heart chambers.^82^

Clinically, the presentation and the long-term prognosis is strictly related to the comorbidities that led to CIED implantation [i.e. for example severe left-ventricular systolic dysfunction in case of implantable cardioverter defibrillator (ICD)] and the severity of TR.

However, if needed in case of severe TR, the therapeutic management must take into account the presence, the location, and the interaction with the TV apparatus of the lead.

Recently, transcatheter edge-to-edge repair with the TriClip system has been demonstrated to be safe and effective in selected TR patients with transvalvular CIED, without affecting CIED function, and TR patients with CIED experienced similar TR reduction and quality-of-life improvements as subjects without CIED.^83^

Nonetheless, from an electrophysiology standpoint, several unresolved questions remain. For instance, the long-term implications of lead jailing, particularly for ICD leads, require further investigation. Additionally, the effectiveness and impact of alternative valve-sparing pacing strategies—such as leadless pacemakers or coronary sinus leads—used in cases where pacing is required after transcatheter valve replacement or in patients with moderate to severe TR prior to pacemaker implantation, remain unclear. A deeper understanding of these issues will be crucial, especially if such therapies are to be approved and expanded beyond inoperable patients.

## Primary tricuspid regurgitation

Organic or primary TR, either acquired or congenital, is caused by a pathological process affecting any of the elements of the TV apparatus. It may present in the form of a pure myxomatous disease, with limited or diffuse leaflet prolapse, or as a flail with a wide range of causes, in particular post-traumatic, caused by endocarditis or following lead extraction. While historically considered a rare aetiology of TR, data from *ad hoc* imaging studies have lately reported a non-negligible prevalence of tricuspid valve prolapse (TVP), particularly in the context of concomitant left-side degenerative mitral regurgitation (MR). In particular, a recent cardiac magnetic resonance-based study showed that TVP was a frequent finding among patients with all grades of organic MR severity and more often associated with advanced TR severity compared with patients without TVP.^84^ Moreover, among a large cohort of severe degenerative MR patients evaluated with systematic transoesophageal screening, concomitant TVP was the most common TR aetiology with a noticeable prevalence of 55%,^85^ in line with both early and recent studies reporting similar percentages (from 13% up to 63%) of TVP in patients with MVP.^84,86,87^ Of note, in this study, compared with the other TR groups, patients with organic TR presented with a lower burden of comorbidities, a less severe clinical presentation, and a lower prevalence of hemodynamically relevant TR and heart chamber remodelling; they had the lowest rate of concomitant TV surgical intervention. While recently the safety and efficacy of percutaneous TR repair with Triclip has been confirmed even in the context of organic TR,^88^ further studies to focus on TR severity progression and long-term outcome according to this aetiology are eagerly awaited to improve our clinical decision-making.

## Data Availability

NA according to the type of manuscript.
